# Antimicrobial susceptibility and multilocus sequence typing of *Clostridium perfringens* isolated from yaks in Qinghai-Tibet plateau, China

**DOI:** 10.3389/fvets.2022.1022215

**Published:** 2022-10-17

**Authors:** Dan Wu, Runbo Luo, Ga Gong, Lihong Zhang, Jiaqi Huang, Chongzhen Cai, Yupeng Li, Irfan Irshad, Rende Song, Sizhu Suolang

**Affiliations:** ^1^College of Animal Science, Tibet Agricultural and Animal Husbandry University, Nyingchi, China; ^2^College of Veterinary Medicine, Gansu Agricultural University, Lanzhou, China; ^3^Pathobiology Section, Institute of Continuing Education and Extension, University of Veterinary and Animal Sciences, Lahore, Pakistan; ^4^Animal Disease Prevention and Control Centre of Yushu Tibetan Autonomous Prefecture, Yushu, China

**Keywords:** yak, antibiotic resistance, multilocus sequence typing, *clostridium perfringens*, toxin genes

## Abstract

*Clostridium perfringens (C. perfringens)* is an opportunistic pathogen that cause necrotic enteritis, food poisoning and even death in animals. In this study, we explored the prevalence, antibiotic resistance and genetic diversity of *Clostridium perfringens* isolated from yak in the Qinghai-Tibet plateau, China. A total of 744 yak fecal samples were collected and assessed for toxin genes, antimicrobial susceptibility and multilocus sequence typing (MLST). Results indicated that 144 out of 744 (19.35%) yak fecal samples were tested to be positive for *C. perfringens*, 75% (*n* = 108, 108/144) were *C. perfringens* type A, 17.36% (*n* = 25, 25/144) were *C. perfringens* type C, 2.78% (*n* = 4, 4/144) were *C. perfringens* type D, and 4.86% (*n* = 7, 7/144) were *C. perfringens* type F. In addition, 2.78% (*n* = 4, 4/144) of the isolates were positive for *cpb2* toxin gene. Antimicrobial susceptibility testing revealed that 98.61% (142/144) of the isolates showed multiple-antibiotic resistance. According to MLST and phylogenetic tree, 144 yak-derived *C. perfringens* isolates had an average of 12.95 alleles and could be divided into 89 sequence types (STs) and clustered in 11 clonal complexes (CCs). The most of isolates belong to type A with a considerable genetic diversity, having Simpson index up to 0.9754. MLST and phylogenetic analysis showed that the isolates under the same clade came from multiple regions. Cross-transmission among isolates and interconnectedness were observed in the genetic evolution. According to the study, the most of the isolates exhibited broad-spectrum antibacterial resistance, diverse alleles, and multiple lethal toxin genes of *C. perfringens*.

## Introduction

*Clostridium perfringens* is an anaerobic and ubiquitous gram-positive pathogen, which normally inhabit the intestines of animals and humans, as well as in the natural environment such as forage grass, soil, excrement and decaying vegetation (1). It is not only cause severe enterotoxaemia and high mortality in ruminants but also contaminates slaughtering and processing chain, which poses a great threat to food safety (2). The pathogenicity of *C. perfringens* can induce acute/sudden death by producing different lethal toxins and enzymes, resulting in multiple diseases, i.e., diarrhea, necrosis, bacteremia and food poisoning (3, 4). Based on the different secreted toxins, *C. perfringens toxins*, mainly categorized into 7 subtypes: CpA (α), CpB (α, β, ε), CpC (α, β), CpD (α, ε), CpE (α, ι), CpF (α, CPE) and CpG (α, NetB) (5). Among them, type A is the most common type. Additionally, lethal toxin β2 (cpb2) is secreted by various types of *C. perfringens* and cause many diseases (6).

Antibiotics have been widely used to treat necrotizing enteritis caused by *C. perfringens* and promote the growth of livestock (7). However, the abuse of antibiotics which causes a serious threat for public health due to multidrug-resistant bacteria and antibiotic-residues in food. It has been reported that the multidrug resistance of *C. perfringens* showed different prevalence in various types of animals around the world, such as in Thailand (8), Korea (9), Pakistan (10), Bangladesh (11), and China (12). However, little is known about the prevalence and antibiotic resistance of *C. perfringens* in yak. Therefore, it is of great significance to investigate the antibiotic susceptibility of *C. perfringens* of yak products in China for effectively controlling the dissemination of *C. perfringens*.

With the development of molecular biology and genomics, many molecular typing techniques are extensively applied to assess bacterial diversity and the prevalence rate of *C. perfringens* (13). According to sequence analysis, Multilocus sequence typing (MLST) is a molecular typing method that combines bioinformatics and high-throughput sequencing. It is a biological method which often used for characterizing bacteria and is considered the gold standard way for bacterial typing (14). As an effective method to solve bacterial population genetics, MLST was performed based on PCR-amplified housekeeping genes (typically 7–10) for generating different sequence types following the permutation and combination of different alleles (15, 16). Hence, MLST is increasingly be used as a tool for strain comparisons and has become a crucial method for molecular epidemiological studies of bacteria.

Yak (*Bos grunniens*) plays a vital role in the country's economy and are a common source of meat, milk, fur and leather for local herders (17). However, yaks are mainly grazed, resulting in various infectious and non-infectious diseases. Among various bacteria, *C. perfringens* is regarded as one of the main reason for morbidity and mortality. Also, it is an emerging challenge for the yak industry. However, research regarding the prevalence, antibiotic resistance and molecular typing of *C. perfringens* of yaks remains scarce to date. Here, we investigated the prevalence, toxin genes, antimicrobial resistance and genetic diversity of *C. perfringens* isolated from yak fecal in Qinghai-Tibet Plateau of China. This article provides epidemiological evidence of *C. perfringens* in Qinghai-Tibet Plateau of China for the prevention and treatment of yak-related diseases caused by this bacterium.

## Materials and methods

### Sample collection

**Figure 1:**
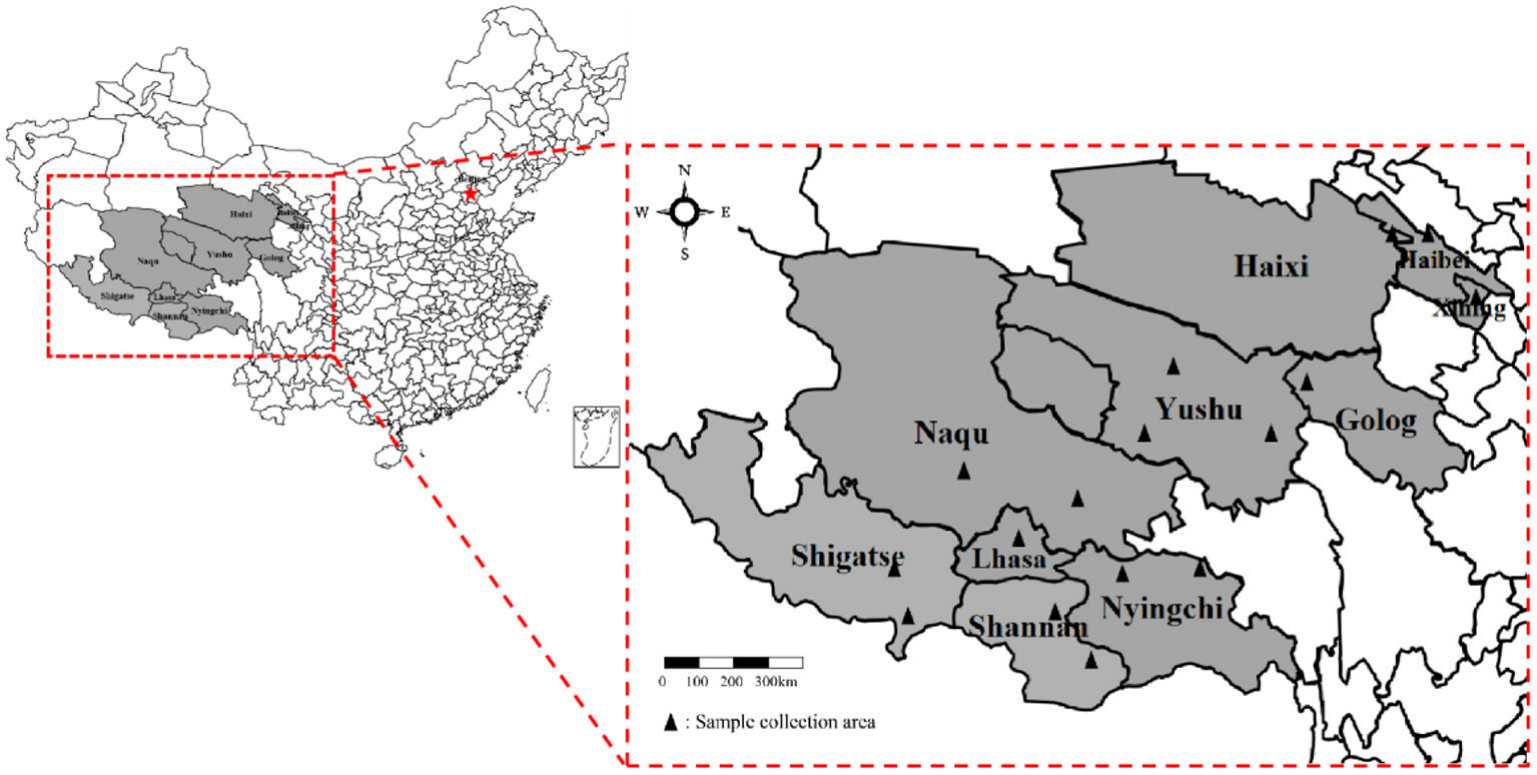
Geographical locations from where samples were collected (Marked as: ▴).

From July 2018 to July 2021, 744 fresh yak fecal samples were collected from the main grazing areas in Qinghai-Tibet Plateau, China. As shown in Figure 1 the information of samples was collected from 13 different cities. Fresh yak feces were collected using sterile fecal swabs, placed at 4°C in an in-vehicle refrigerator, and sent to the Laboratory of Preventive Veterinary of Tibet Agriculture and Animal Husbandry University.

### Isolation and culture of *C. Perfringens*

1 g of content was collected into a tube containing 9 ml of sterile phosphate-buffered saline (PBS, pH 7.4), and subsequently they were inoculated onto fluid thioglycollate medium (FTG) (Hopebio, Qingdao, China). Inoculated medium was cultured under anaerobic condition at 41°C for 18~24 h. After purification culture, the colonies were inoculated onto blood agar medium with 5% defibrinated sheep blood (Hopebio, Qingdao, China) and cultured in the anaerobic pack (Mitsubishi, Tokyo, Japan) at 37°C for 18–24 h under anaerobic condition. Then, single colonies with a hemolytic zone were analyzed according to the bacterial shape, color, gram staining smears and growth features as described by previous study (18). The isolates of *C. perfringens* were selected from each positive sample for toxin gene detection, susceptibility test, MLST and phylogenetic analysis.

### DNA extraction

The single purified colonies were inoculated in RCM, sealed with paraffin, and incubated at 41°C till *D*_600nm_ = 0.6~0.8. Then DNA was extracted by TIAN amp Bacteria DNA Kit (TIANGEN, Beijing, China) and stored at −20°C.

### Detection of *C. Perfringens* toxin gene by PCR amplification

Relevant primers were synthesized according to the previous literature (19, 20). The 16S rRNA gene, common lethal toxin genes (*cpa, cpb, etx, iap, cpe, netB*) and beita2 toxin gene (cpb2) were examined for each *C. perfringens* isolate using PCR or multiplex PCR techniques (Table 1). Each PCR amplification was conducted in a reaction volume of 50 μL, including 1 μL of upstream primer, 1 μL of downstream primer, 25 μL of 2× Tap-T DNA Polymerase (TIANGEN, Beijing, China), 2 μL of DNA template, and 21 μL of ddH_2_O. Subsequently, the PCR products were electrophoresed on a 1.5% agarose gel, photographed and analyzed by a gel documentation system (Tanon, Shanghai, China). The products were purified by TIAN gel Midi Purification Kit (TIANGEN, Beijing, China), sequenced by Tsingke Biotechnology Co., Ltd. (Chengdu) and further identified by NCBI website (https://www.ncbi.nlm.nih.gov/). Finally, each isolate was divided into seven (A~G) toxin types.

**Table 1:** PCR primer information.

**Gene name**		**Primer sequence**	**Fragment**
		**(5′–3′)**	**size(bp)**
16S rDNA	F	AGAGTTTGATCCTGGCTCAG	1465
	R	GGTTACCTTGTTACGACTT	
*cpa*	F	GCTAATGTTACTGCCGTTGA	325
	R	CCTCTGATACATCGTGTAAG	
*cpb*	F	GCGAATATGCTGAATCATCTA	196
	R	GCAGGAACATTAGTATATCTTC	
*etx*	F	GCGGTGATATCCATCTATTC	656
	R	CCACTTACTTGTCCTACTAAC	
*iap*	F	ACTACTCTCAGACAAGACAG	443
	R	CTTTCCTTCTATTACTATACG	
*cpe*	F	GGAACCCTCAGTAGTTTCAAGT	461
	R	CTGTAGCAGCAGCTAAATCAAG	
*netB*	F	GGAAGGCAACTTTAAGTGGAAC	680
	R	GTTTGTTCCTCGCCATTGAGT	
*cpb2*	F	ATGAAAAAAATTATTTCAAAGTTTAC	798
	R	CTATGCACAATACCCTTCACCAAA	

### Antimicrobial susceptibility test

The susceptibility of the isolates to 26 antimicrobials was determined based on the Kirby-Bauer disc diffusion method suggested by the CLSI (21). Single colonies were inoculated in RCM with paraffin liquid seal and kept at 41°C. When *D*_600nm_ = 0.6~0.8, 300 μL of supernatant bacterial solution was drawn and spread evenly on Mueller-Hinton agar (MHA) (Hopebio, Qingdao, China), and 26 antibiotic susceptibility discs (Microbial Reagent, Hangzhou, China) were placed in the medium (4 discs in each medium), cultured anaerobically at 41 °C for 24 h. The antimicrobials used were all drugs commonly used in livestock and humans, including penicillin (10 UI), oxacillin (10 μg), ampicillin (10 μg), cephalexin (30 μg), cefazolin (30 μg), cefotaxime (30 μg), cefuroxime (30 μg), ceftazidime (30 μg), gentamicin (10 μg), kanamycin (30 μg), streptomycin (10 μg), tetracycline (30 μg), doxycycline (30 μg), minocycline (30 μg) erythromycin (15 μg), madicin (30 μg), ofloxacin (5 μg), ciprofloxacin (5 μg), bacitracin (10 UI), polymyxin B (300 μg), florfenicol (30 μg), chloramphenicol (30 μg), chloramphenicol (30 μg), sulfamethoxazole (300 μg), clindamycin (2 μg), furazolidone (300 μg), vancomycin (30 μg). The types of antibacterial drugs and determination criteria for drug resistance were shown in Supplementary Table 1. Finally, the diameter of the inhibition circle was measured, and the resistance of the isolates was determined according to the *Performance Standards for Antimicrobial Susceptibility Testing* by CLSI and the manufacturer's instructions (Microbial Reagent, Hangzhou, China).

### Housekeeping gene PCR amplification

According to Jost et al. (22), eight housekeeping genes (*plc, ddlA, dut, glpK, gmk, recA, sod* and *tpi*) of *C. perfringens* were selected to amplify the DNA of the isolates by PCR (Table 2). The PCR products were electrophoresed on a 1.5% agarose gel, recovered by the TIAN gel Midi Purification Kit. A nucleic acid protein detector measured the DNA concentration and purity. The recovered DNA fragments were ligated into the pMDTM18-T vector overnight at 4°C using the pMD^TM18^-T Vector Kit (TaKaRa, Tokyo, Japan) and transformed into DH5α competent cells (TaKaRa, Tokyo, Japan) and screened using LB medium containing Amp^+^. For the bacterial solution identified as positive by PCR, the plasmid was extracted following the instructions of the High Pure Maxi Plasmid Kit (TIANGEN, Beijing, China) and sequenced by Tsingke Biotechnology Co., Ltd. (Chengdu).

**Table 2:** Multilocus sequence typing primer information.

**Gene name**		**Primer sequence**	**Fragment**
		**(5′–3′)**	**size/bp**
*plc*	F	ATATGAATGGCAAAGAGGAAAC	544
	R	AGTTTTTCCATCCTTTGTTTTG	
*ddlA*	F	ATAATGGGGGATCATCAGTTGC	429
	R	TTATTCCTGCTGCACTTTTAGG	
*dut*	F	TTAAGTATTTTGATAACGCAAC	441
	R	CTGTAGTACCAAATCCACCACG	
*glpK*	F	TGGGTTGAGCATGATCCAATGG	574
	R	CACCTTTTGCTCCAAGGTTTGC	
*gmk*	F	TAAGGGAACTATTTGTAAAGCC	475
	R	TACTGCATCTTCTACATTATCG	
*recA*	F	GCTATAGATGTTTTAGTTGAGG	475
	R	CTCCATATGAGAACCAAGCTCC	
*sod*	F	GATGCTTTAGAGCCATCAATAG	478
	R	AATAATAAGCATGTTCCCAAAC	
*tpiA*	F	AAATGTGAAGTTGTTGTTTGCC	451
	R	CATTAGCTTGGTCTGAAGTAGC	

### Multilocus sequence typing and phylogenetic analysis

The raw sequencing of 8 housekeeping genes (*ddlA, dut, glpK, gmk, plc, recA, sod, tpiA*) of 144 strains of *C. perfringens* was proofread spliced and cut. The processed housekeeping gene sequences were then assigned allele numbers using BIONUMERICS 8.1 (Applied Maths, Keistraat, Belgium) software, and sequence type (ST) was given to each strain. The eight allele numbers of the isolates were formed into profiles, and the similarity coefficients were calculated by the categorical method to construct a minimum spanning tree (MST). In addition, strains with 7 or more identical loci out of 8 loci were defined as the same Clonal Complex (CC). Similarity coefficients were calculated by the definite method, and clustering trees were built by the Unweighted Pair Group Method Arithmetic means (UPGMA) method for evolutionary genetic analysis. Finally, the polymorphism of allelic loci of each housekeeping gene and strains in different regions were quantified using Simpson Diversity Index (Ds).

## Results

### Prevalence of *C. perfringens*

The results showed that 144 samples (19.35%) were positive for *C. perfringens*. Different regions' positive rates of *C. perfringens* were shown in Table 3, including 61 (23.12%, 61/264) positive samples in Qinghai province and 83 (17.29%, 83/480) positive samples in Tibet. 144 isolates were totally acquired in all samples for subsequent experiments.

**Table 3:** Isolation rates of *Clostridium perfringens* in samples from different regions.

**Province**	**Sampling**	**Sampling**	**No. of**	**No. of**	**Separation**
	**time**	**location**	**samples**	**isolates**	**rate**
Qinghai	2018.10	Qilian County	18	7	38.89%(7/18)
	2018.07	Chengduo County	19	3	15.79%(3/19)
	2018.10	Qingshuihe Town	45	9	20%(9/45)
	2019.07	Datong County	82	18	21.95%(18/82)
	2020.10	Zhiduo County	52	12	23.08%(12/52)
	2020.10	Zaduo County	48	12	25%(12/48)
	Total 1		264	61	23.12%(61/264)
Tibet	2020.07	Baingoin County	120	22	17.69%(22/120)
	2020.09	Lhasa	76	12	15.79%(12/76)
	2020.09	Shigatse	20	7	35%(7/20)
	2021.05	Nyingchi	60	13	21.67%(13/60)
	2021.07	Qusong County	66	10	15.15%(10/66)
	2021.07	Sangri County	62	10	16.13%(10/62)
	2021.07	Lhari County	76	9	11.76%(9/76)
	Total 2		480	83	17.29%(83/480)
	Total 3		744	144	19.35%(144/744)

### Toxin gene screening

All isolates (*n* = 144) belonging to different virulence types of *C perfringens* are shown in Table 4. The total 108 (75%, 108/144) isolates were assessed to be positive for *C. perfringens* type A, 25 (17.36%, 25/144) were *C. perfringens* type C, 4 (2.78%, 4/144) were *C. perfringens* type D, and 7 (4.86%, 7/144) were *C. perfringens* type F. In addition, 4 isolates (2.78%, 4/144) contained the *cpb2* toxin gene. The *iap* and *netB* genes were not detected in none of the isolates (Supplementary Table 2).

**Table 4:** Distribution of different virulence types of *Clostridium perfringens* isolated from Yaks.

**Toxino**	**Toxic/virulent**	**No. of positive strains in**		
**types**	**genes**	**different regions (%)**		
		**Qinghai**	**Tibet**	**Total**
		**(*n* = 61)**	**(*n* = 83)**	**(*n* = 144)**
A	*cpa*	48 (78.69%)	56 (67.47%)	104 (72.22%)
	*cpa + cpb2*	2 (3.28%)	2 (2.41%)	4 (2.78%)
C	*cpa + cpb*	5 (8.20%)	20 (24.10%)	25 (17.36%)
D	*cpa + etx*	4 (6.56%)	0	4 (2.78%)
F	*cpa + cpe*	2 (3.28%)	5 (6.02%)	7 (4.86%)

### Antibiotic resistance

**Figure 2:**
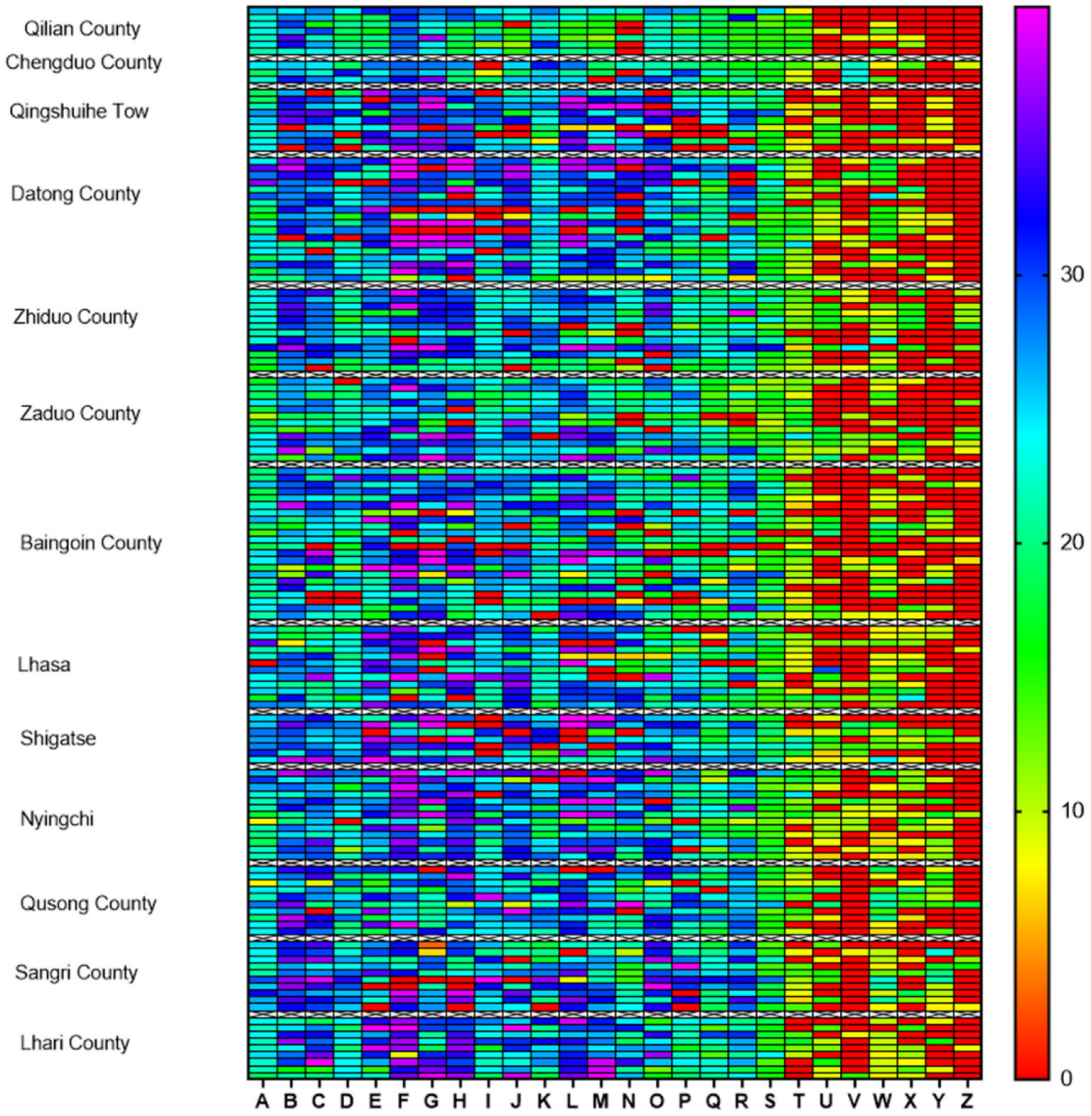
Heat map of antibiotic resistance of 144 *Clostridium perfringens* isolates from the Qinghai-Tibet Plateau. The different regions are listed on the left vertical axis and antibiotics (A–Z) are marked on the horizontal axis. Colored scale bars represent the zone of inhibition ranges between 0 and 40 mm. (A) Vancomycin; (B) Minocycline; (C) Doxycycline; (D) Ofloxacin; (E) Cefotaxime; (F) Cefazolin; (G) Ampicillin; (H) Cefuroxime; (I) Chloramphenicol; (J) Ceftazidime; (K) Florfenicol; (L) Penicillin; (M) Cephalexin; (N) Oxacillin; (O) Tetracycline; (P) Ciprofloxacin; (Q) Mideamycin; (R) Clindamycin; (S) Furazolidone; (T) Bacitracin; (U) Gentamicin; (V) Polymyxin B; (W) Erythromycin; (X) Kanamycin; (Y) Sulfamethoxazole; (Z) Streptomycin.

**Figure 3:**
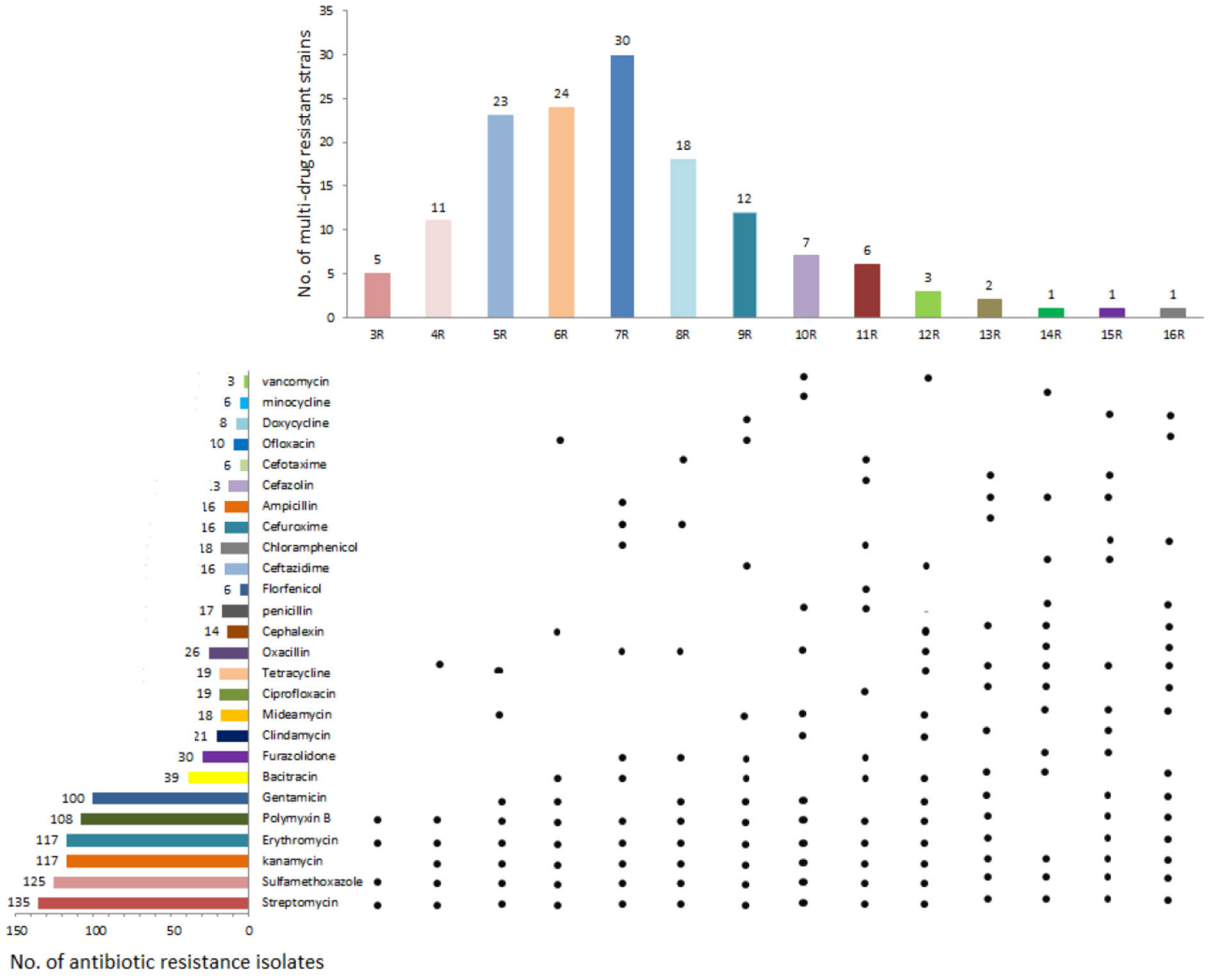
Drug resistance of 144 yak-derived *Clostridium perfringens* in Qinghai-Tibet Plateau. The left side is the number of resistant strains of the corresponding antibiotics; the right side are the Isolates in corresponding antibiotic resistance profiles showing resistance to this antibiotic, the top is the number of isolates resistant to multiple antibiotics and the bottom is the main yak-derived isolates in antibiotic resistance spectrum.

The isolated strains in this study displayed broad antibiotic resistance. The differences in the similarities of antibiotic resistance of isolates from various regions were observed by the heat map (Figure 2). The antibiotics with the highest amount of resistance were streptomycin (93.75%, 135/144), followed by sulfamethoxazole (86.81%, 125/144), kanamycin (81.25%, 117/144), erythromycin (81.25%, 117/144), polymyxin B (75%, 108/ 144), and gentamicin (69.44%, 100/144). The most of the isolates exhibited susceptivity to vancomycin, minocycline, cefotaxime, florfenicol, and doxycycline (Figure 3).

**Figure 4:**
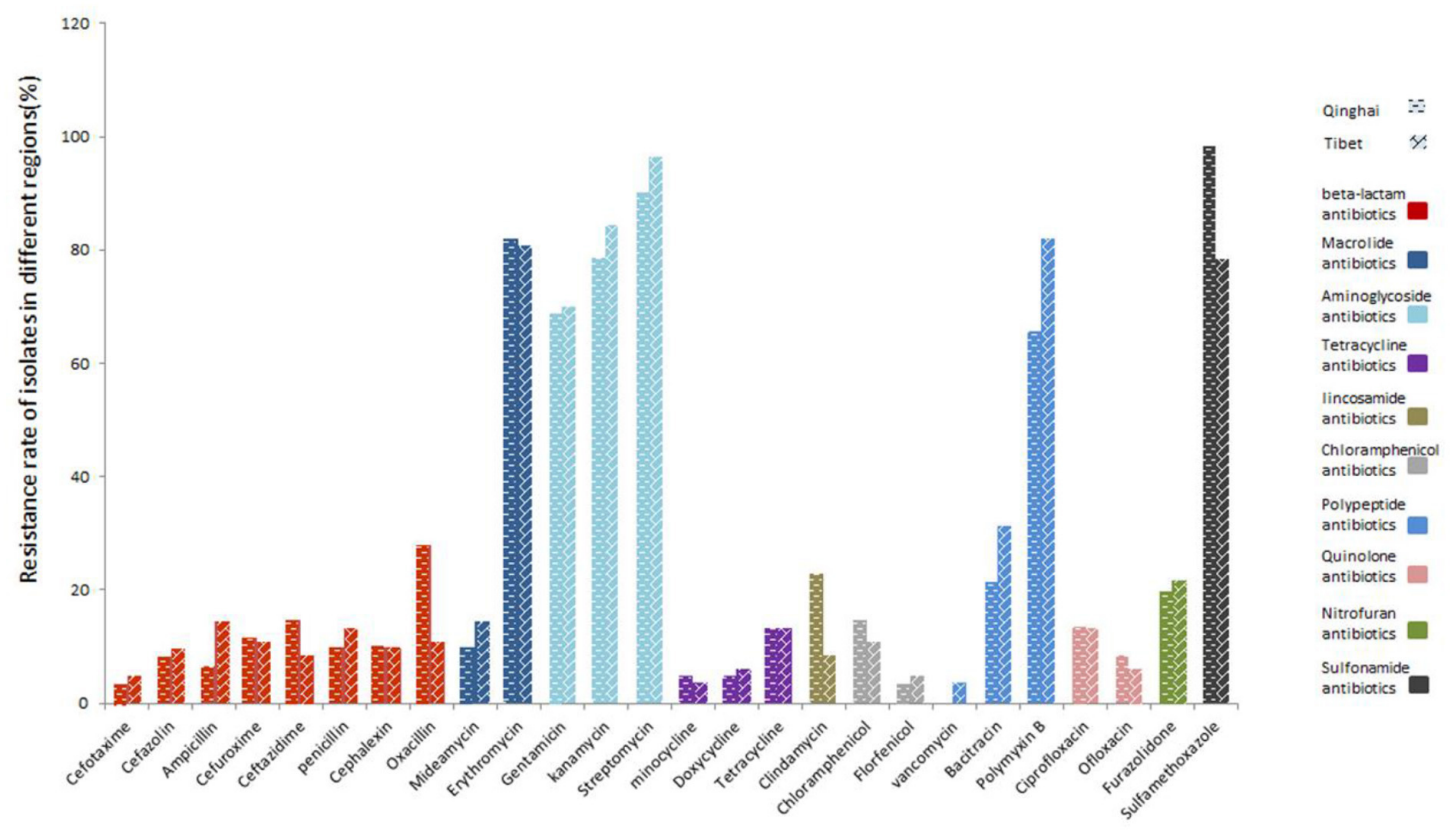
Resistance of yak-derived *Clostridium perfringens* to different antibiotics in different regions.

There is a difference in the antibiotics against *C perfringens* isolates in different regions (Figure 4). Resistance against aminoglycoside antibiotics (gentamicin, kanamycin, streptomycin) and sulfonamide antibiotics (sulfamethoxazole) were more than 65% in both Tibet and Qinghai. Moreover, the percentage of strains isolates from Tibet displayed the highest resistance to aminoglycoside streptomycin (96%), while isolates from Qinghai showed the highest resistance to sulfamethoxazole antibiotics (98%). Among the macrolide antibiotics, the resistance against erythromycin was higher (82% in Qinghai and 81% in Tibet) and lowered against midecamycin (10% in Qinghai and 14% in Tibet). Besides, the isolates from Tibet and Qinghai showed low resistance to β-lactam, tetracycline, chloramphenicol, quinolone and nitrofuran antibiotics.

The percentage of multidrug-resistant isolates was 98.61% (142/144; Supplementary Table 3). Most *C. perfringens* were resistant to four types of antibiotics, accounting for 29.17% (42/144) of the isolated strains. Of which 77 isolates (53.47%, 77/144) were resistant to 5–7 antibiotics. Two isolates showed resistance to nine types of antibiotics.

### ST and minimum spanning tree analysis

The polymorphism of *gmk* gene was the highest (*n* = 50), and the lowest was the *glpK* gene (*n* = 15). The average number of alleles for all loci was 12.95. According to the analysis, the allelic genetic diversity of the isolates showed 0.5068 ≤ Ds ≤ 0.9560, and the proportion of polymorphism for *gmk* gene was the highest, while *tpi* gene was the lowest. In addition, according to the sequence types (STs) of *C. perfringens* and eight different housekeeping genes and the allelic profiles of the corresponding strains, 144 strains of *C. perfringens* were classified into 89 STs. Among that the most prolific ST was ST76 (9.72%, 14/144), followed by ST10 (6.25%, 9/144), ST3 (3.47%, 5/144), ST33 (2.78%, 4/144) (Table 5). All other STs (ST4, ST8, ST15, ST17, ST25, ST56, ST63, ST81) and (ST12, ST16, ST22, ST36 ST12, ST16, ST22, ST36, ST38, ST41, ST45, ST47, ST73, ST80, ST89) contained 3 (2.08%, 3/144) and 2 (1.39%, 2/144) strains; respectively. The other STs only contain a single strain (0.69%, 1/144). All ST76 contained isolates from Tibet (Nyingchi, *n* = 1; Qusong County, *n* = 7; Sangri County, *n* = 6). ST10 contained five strains from Qinghai Province (Qingshuihe Tow, *n* = 2; Datong County, *n* = 3) and four from Tibet (Lhasa, *n* = 1; Shigatse, *n* = 3). All ST3 contains strains from Qilian County, Qinghai Province. All ST33 contains strains from Zaduo County, Qinghai Province.

**Table 5:** Genes, fragment size, number of alleles, average number of alleles and simpson diversity index (Ds) of yak-derived *Clostridium perfringens* strains.

**Genes**	***plc***	***ddlA***	***dut***	***glpK***	***gmk***	***recA***	***sod***	***tpi***
Fragment size/bp	544	429	441	574	475	475	478	451
Number of alleles	38	43	41	15	50	26	46	16
Average number of alleles	15.12	15.78	16.89	3.72	23.14	8.21	18.11	2.60
Simpson Diversity index (Ds)	0.9255	0.9344	0.9338	0.7542	0.9560	0.8867	0.9424	0.5068

### Phylogenetic and genetic diversity analysis

**Figure 5:**
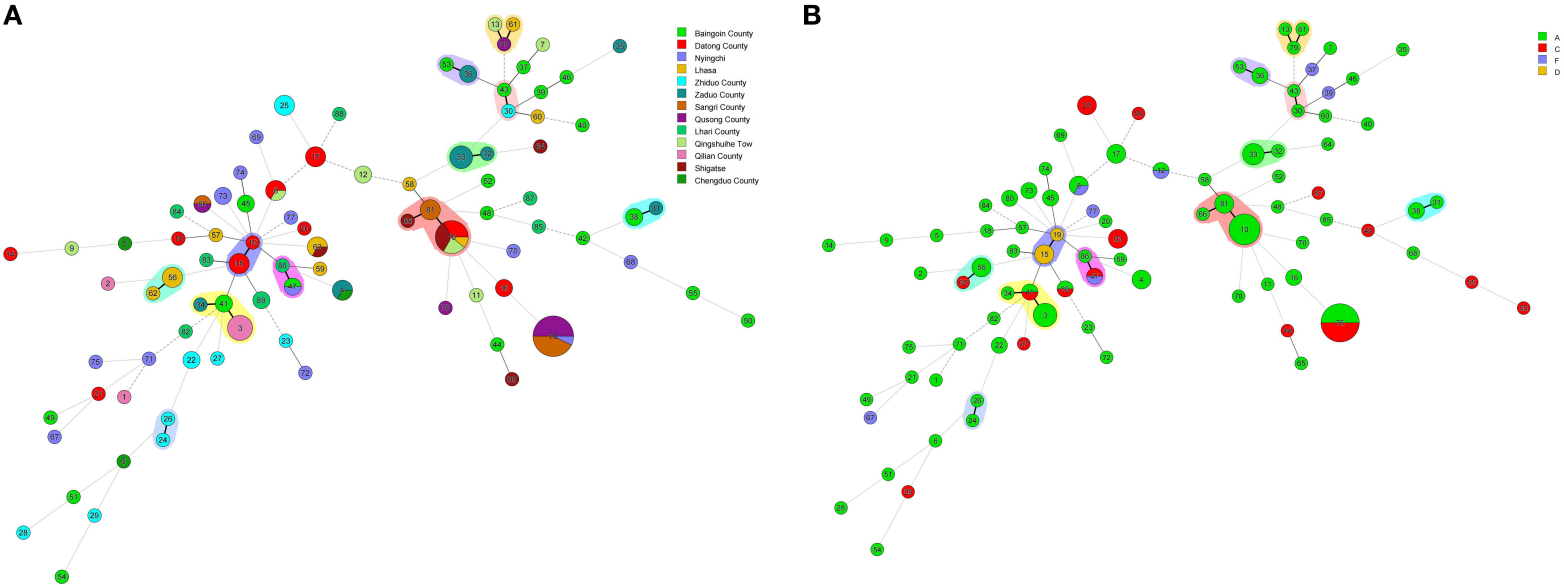
**(A)** Minimal evolutionary tree generated with different Source characteristics. **(B)** Minimal evolutionary tree generated with different Toxin types as features. A circle represents a sequence type, the size of the circle represents the number of strains, the number on the circle represents the name of the ST type. The different connecting lines between the two circles represent the difference between the two ST types, the thick solid line indicates that there is one locus difference between the two ST types, the thin solid line indicates that there are two or three different loci, the thin dotted line indicates that there are four different loci, and the remaining lines indicates more than four different loci. Strains in the same shade represent the same Clonal complex (CC).

The minimum spanning tree mainly consisted of 11 clonal complexes (CC1–CC11), accounting for 35.42% (51/144) of all isolates. As shown in Figures 5A,B, CC1 contained 3 (2.08%, 3/144) strains (ST13, ST61 and ST79), CC6 contained 13 (9.03%, 13/144) strains (ST10, ST81 and ST66), and CC10 contained 8 (5.56%, 8/144) strains (ST3, ST34 and ST41). The other CCs contained only 2 strains (5.56%, 8/144). As per the differences in alleles, the evolutionary relationship among CC1, CC2, and CC3 was genetically close, and CC7, CC8, and CC10 was genetically belonging to the same clade. However, CC4, CC6, and CC9 were the furthest.

**Figure 6:**
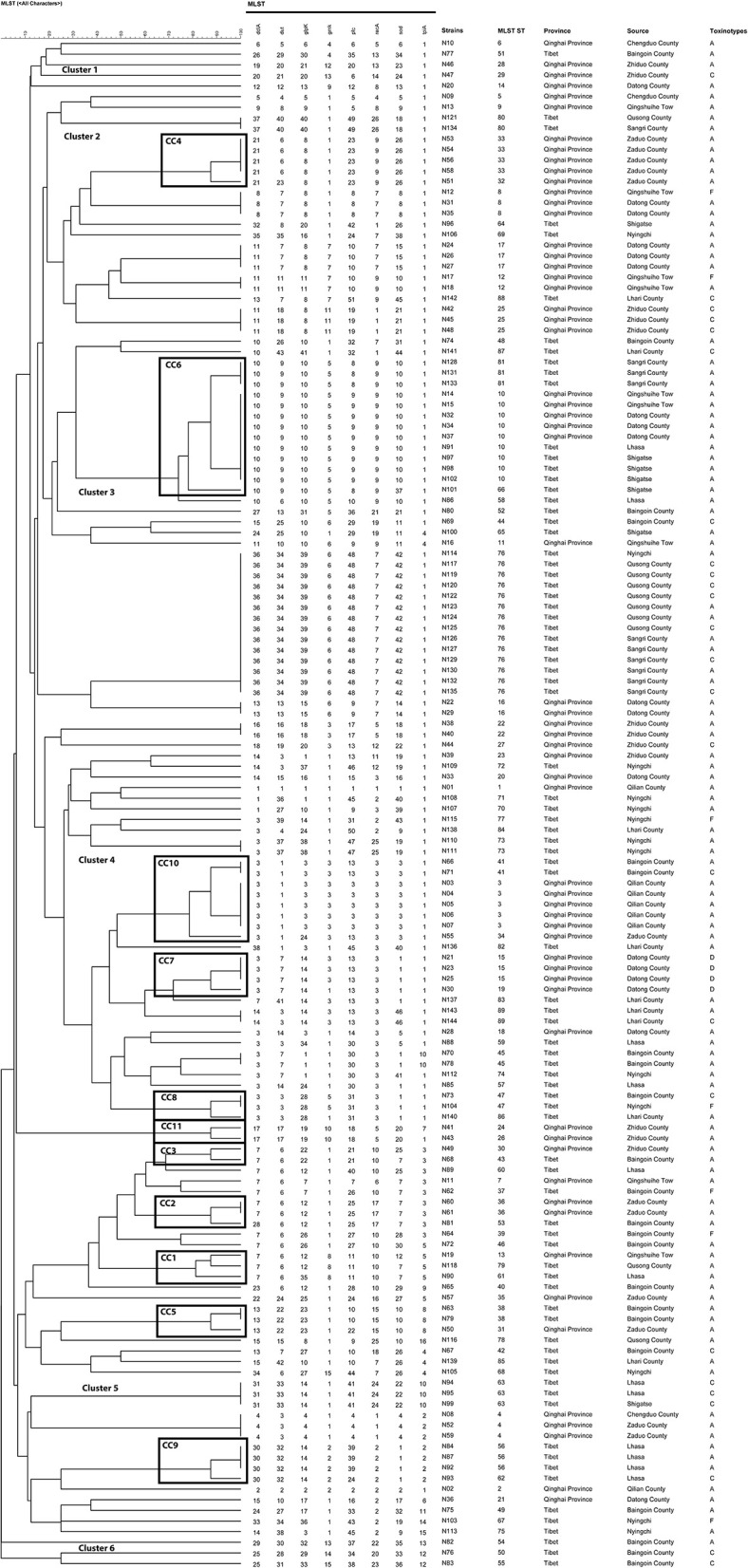
Phylogenetic tree and allelic profiles of 144 yak-derived *Clostridium perfringens* sequence types (STs).

In general, the isolates of CC were clustered together, which demonstrated the significant cluster of the tree, and the same was noticed with the minimum spanning tree. The phylogenetic tree reflected the differences between different isolates. As shown in Figure 6, the isolates under the same clade were from multiple regions. They were evenly distributed in toxin typing but not related, which indicated that yak-derived *C. perfringens* on the Tibetan plateau had cross-transmission and connection in its genetic evolution. The evolutionary relationship between ST-50, ST-54, ST-55, and other STs was the farthest for ST type. The results in the phylogenetic tree and the minimum spanning tree were consistent. There is a difference that the isolates from the same branch were from different regions, whereas the evolution in some areas (Qusong and Sangri Counties) was relatively close and clustered to the same clade.

To better understand the genetic diversity of the isolates, the Simpson index (Ds) was employed to calculate the genetic diversity. A total of 114 isolates were divided into 89 STs with Ds = 0.9754. The genetic diversity of Qinghai and Tibet was close, 61 isolates in Qinghai were divided into 36 STs (Ds = 0.9594) and 83 isolates in Tibet were divided into 54 STs (Ds = 0.9551). The highest genetic diversity was found in Baingoin County (22 isolates were divided into 19 STs, Ds = 0.9421), followed by Nyingchi (13 isolates were classified into 12 STs, Ds = 0.9112), Datong County (18 isolates were classified into 10 STs, Ds = 0.8765). The least genetic diversity was observed in Qilian County (7 isolates were divided into 3 STs, Ds = 0.4490), followed by Qusong County (10 isolates were divided into 4 STs, Ds = 0.4800), Sangri County (10 isolates were divided into 3 STs, Ds = 0.5400). The remaining areas in order of genetic diversity richness were Lhari County (Ds = 0.8642), Lhasa (Ds = 0.8611), Zhiduo County (Ds = 0.8611), Zaduo County (Ds = 0.8056), Shigatse (Ds = 0.7347), and Chengdu County (Ds = 0.6667), Qingshuihe Tow (Ds = 0.5926). The area with the highest genetic diversity was Baingoin County (22 isolates were divided into 19 STs, Ds = 0.9421), followed by Nyingchi (13 isolates into 12 STs, Ds = 0.9112), Datong County (18 isolates into 10 STs, Ds = 0.8765). The area with the lowest genetic diversity was Qilian County (7 isolates were divided into 3 STs, Ds = 0.4490), followed by Qusong County (10 isolates into 4 STs, Ds = 0.4800), Sangri County (10 isolates into 3 STs, Ds = 0.5400). The remaining areas are Lhari County (Ds = 0.8642), Lhasa (Ds = 0.8611), Zhiduo County (Ds = 0.8611), Zaduo County (Ds = 0.8056), Shigatse (Ds = 0.7347), Chengduo County (Ds = 0.6667), Qingshuihe Tow (Ds = 0.5926), in descending order of genetic diversity.

## Discussion

In this study, the positive rate of *C. perfringens* was 19.35% (144/744), which is lower than that of 45% of Tibetan sheep in Gansu and Qinghai regions of China (23), 65.42% of cattle in Pakistan (24), 69.7% cattle and 61.5% in Egypt (25), closing to the cattle (20.8%) and goats (18.3%) in northeast India (26). Moreover, several studies have shown the varying prevalence of *C. perfringens* in chickens, pigs, poultry, buffalo, and sheep (27, 28). Although *C. perfringens* mainly colonize the intestine, its positive rate is subject to the living environment of the host animal. In the current study, the positive rate of *C. perfringens* from yak was lower than that of animals from other regions. The reason may be the long been living on the Tibetan plateau with high altitude, low temperature, low humidity, strong UV light, few airborne particulates and bacterial vectors, that prohibit bacteria from reproduction and transmission (29). This is in line with the microbial diversity and physicochemical characteristics of soil at high altitudes, where gram negative bacteria proliferate more as compare to gram positive bacteria (30).

In this study, genotyping results showed that the isolated sample were identified as *C. perfringens* types A, C, D and F, which suggesting the genotypic diversity of yak *C. perfringens*. The *cpa, cpb, etx, cpe* and *cpb2* toxin genes were observed from yak feces, while *iap* and *netB* genes were not detected in samples. A number of researches showed that *C. perfringens* type A is a major category associated with food poisoning (31, 32). Consistent with previous reports, 108 (75%, 108/144) isolated strains were identified as *C. perfringens* type A with a relatively high positive rate, indicating that *C. perfringens* type A come as a potential source of infection for humans and animals. *C. perfringens* type C contained *cpa* and *cpb* toxin genes, of which *cpb* is the key pathogenic factor for this bacterium with cytotoxic and lethal activity (33). A total of 25 (17.36%, 25/144) isolates were identified as *C. perfringens* type C, which often causes diarrhea, necrotizing enterocolitis, enterotoxemic and sudden death in yaks. In addition, the study detected *C. perfringens* types D and F for the first time in the yak fecal samples. A total of 4 (2.78%) of *C. perfringens* isolates were type D, with *cpa* and *etx* toxins. It is reported that *C. perfringens* type D can cause enterotoxemic in cattle, sheep, and goats, which may cause sudden death in sheep and goats (34). Previous studies indicated that the *Enterotoxin gene (cpe)* is related to intestinal diseases and food poisoning (35). We found that 7 isolates of *C. perfringens* type F carries the *cpe* gene, rating 4.86%, which is in line with the previous study (36). It was worth noting that 2.78% (*n* = 4, 4/144) of the isolates were positive for *cpb2* toxin gene in *C. perfringens* type A. The Beta-2 toxin was encoded by the cpb2 gene, which is associated with gastrointestinal disease in humans and animals (37) and has been found in many animal species such as neonatal calves (38), chickens (39), and piglets (40).

Infectious diseases are important due to their production losses in animals which is a major economic issue (41). In recent years, the misuse of antibiotics may increase gut microbial resistance, especially in some zoonotic pathogens. Numerous studies showed that multi-resistant *C. perfringens* exist in various animals and animal foods, posing a significant concern to public health (42). In this study, we found that the isolates from yak were extensively resistant to 26 antibiotics in Qinghai-Tibet Plateau. The resistance rate of the isolates were as follows: streptomycin (93.75%), sulfamethoxazole (86.81%), kanamycin (81.25%), erythromycin (81.25%), polymyxin B (75%), and gentamicin (69.44%) with susceptibly to vancomycin, minocycline, cefotaxime, florfenicol, and doxycycline. In addition, the isolates showed high multi-resistance rate (98.61%), and 53.47% of the isolates were resistant to at least 5 classes antibiotics. This phenomenon might be due to the antimicrobial resistance of strain from environment. It has been proved that the resistance of *C. perfringens* to macrolides, and the variability in the resistance of different macrolides is primarily due to different resistance mechanisms (43). The related study had shown that *Clostridium* can carry tetracycline resistance genes encoding ribosomes that protect cytoplasmic proteins (44), while bacitracin can effectively treat necrotizing enteritis and reduce morbidity and mortality (45). Moreover, antibiotics of β-lactam have been proven to be effective against *C. perfringens* (46). In the present study, most of the isolates were susceptible to β-lactam, tetracycline, chloramphenicol, quinolone and nitrofuran antibiotics. The misuse of antibiotics may be a predominant cause of the increase in multidrug resistance to antibiotics (47). Hence, considering the rising rate of antimicrobial resistance, it is necessary to constantly monitor the antimicrobial susceptibility of *C. perfringens* from yaks to reduce the trend of resistance for effective prevention and treatment of relevant diseases.

MLST is usually used for classifying population diversity and comparing the genetic evolution of *C. perfringens* in animals, environment and humans. This research examined 15–50 (average 12.95) loci alleles 0.89 STs and 11 CCs were recognized among *C. perfringens* strains. In comparison, Liu et al. (48) examined an average of 13.5 alleles and 41 STs and 4 CCs C perfringens strains from animal and the environment. It is identified that an average of 5.9 alleles, 22 STs and 6 CCs among 61 isolates from necrotic enteritis (NE) and healthy chickens (49). Previous study (50) analyzed 139 isolates from poultry affected by NE, and identified an average of 12.2 alleles, 41 STs and 6 CCs. Xu et al. (41) analyzed 39 isolates from retail chicken products and diseased chickens and identified an average of 12.13 alleles, 29 STs and 3 CCs. In another study (51), 110 isolates were analyzed and identified an average of 16.25 alleles, 74 STs and 7 CCs. In this study, the average number of alleles is not the highest as compared to the previous studies. According to the results, the STs cluster showed the highest number, as determined by the Simpson diversity index i.e., 0.9754 (0.9594 in Qinghai Province and 0.9551 in Tibet), which indicates the considerable genetic diversity of yak-derived *C. perfringens*. In addition, among all STs, ST76 (9.72%, 14/144) had the largest number of isolates, which were all from Tibet, Qusong and Sangri Counties. This may due to the close geographical location, that led to cross-transmission. The CCs with the highest number of strains were CC6, which contains three STs (ST10, ST81 and ST66) with 13 strains (9.03%, 13/144). The strains CC6 was mainly from Qingshuihe Town, Datong County of Qinghai Province and Lhasa, Shigatse, and Sangri County of Tibet, which are widely distributed geographically and the main population area in the Qinghai-Tibet Plateau. Moreover, some strains of CCs were not clustered and were found only in one region, such as CC4 strains in Zaduo County, CC7 strains in Datong County, CC9 strains in Lhasa and CC11 strains in Zhiduo. These strains represent the dominant populations in region. In addition, according to the genetic relationship of the isolates, the isolates came from different regions under the same clade and were evenly distributed in all toxin types without specific relatedness. The above results indicated that there is cross-transmission of *C. perfringens* from Qinghai-Tibetan Plateau, having some link in genetic evolution. In the future, more methods can be adopted to verify this conclusion to find correlations between phylogenetic and biological traits of yak-derived *C. perfringens*.

## Conclusion

In summary, this is the first study that reported the prevalence, characterization of antimicrobial resistance and genetic diversity of yak *C. perfringens* in the Qinghai-Tibet plateau, China. This research showed a relatively high positive rate of *C. perfringens* with broad-spectrum antimicrobial resistance. Genotyping results indicated the presence of different type of *C. perfringens* (types A, C, D, and F), suggesting considerable genetic diversity. Moreover, MLST indicated the cross-transmission among different regions and found evenly distributed toxins without specific relatedness. This demonstrated that antimicrobial-resistance strains of yak origin pose a potential risk toward public health. Therefore, it is necessary to constantly monitor the antimicrobial susceptibility of *C. perfringens* from yaks to reduce and identify the trend of resistance for effective strategies to prevent such issues.

## Data availability statement

The original contributions presented in the study are included in the article/Supplementary material, further inquiries can be directed to the corresponding author.

## Ethics statement

The animal study was reviewed and approved by the Ethical Committee of Tibet Agricultural and Animal Husbandry University.

## Author contributions

DW and RL: conceptualization, formal analysis, writing—original draft, and writing—review and editing. GG and LZ: methodology, validation, and investigation. JH and II: methodology and data curation. CC: data curation and visualization. YL: validation and visualization. RS: investigation, resources, and supervision. SS: conceptualization, project administration, and funding acquisition. All authors contributed to the article and approved the submitted version.

## Funding

This work was supported by the China Agriculture Research System of MOF and MARA (CARS-37); Key Project of Science and Technology Department of Tibet Autonomous Region (201901); graduate innovation program of Tibet Institute of agriculture and animal husbandry (YJS2021-11), and the Science and technology plan of Qinghai Province (Grant No. 2020-QY-218).

## Conflict of interest

The authors declare that the research was conducted in the absence of any commercial or financial relationships that could be construed as a potential conflict of interest.

## Publisher's note

All claims expressed in this article are solely those of the authors and do not necessarily represent those of their affiliated organizations, or those of the publisher, the editors and the reviewers. Any product that may be evaluated in this article, or claim that may be made by its manufacturer, is not guaranteed or endorsed by the publisher.
