# Supplementary material for: Antimicrobial susceptibility and multilocus sequence typing of Clostridium perfringens isolated from yaks in Qinghai-Tibet plateau, China
Source: Front Vet Sci. 2022 Oct 17;9:1022215. doi: 10.3389/fvets.2022.1022215 (PMC9619089; doi:10.3389/fvets.2022.1022215)
Supplement: Supplementary file 1 [file Data_Sheet_1.docx]

**Supplementary Table 1** Types of Antibacterial Drugs and Determination Criteria for Drug Resistance

| Types of antibiotics | Drugs | The size of the zone of inhibition(mm) | | |
| --- | --- | --- | --- | --- |
|  |  | Resistant | Intermediary | Sensitive |
| beta-lactam antibiotics | Cefotaxime | ≤14 | 14-23 | ≥23 |
|  | Cefazolin | ≤14 | 14-18 | ≥18 |
|  | Ampicillin | ≤13 | 13-17 | ≥17 |
|  | Cefuroxime | ≤14 | 14-23 | ≥23 |
|  | Ceftazidime | ≤14 | 14-18 | ≥18 |
|  | penicillin | ≤13 | 13-17 | ≥17 |
|  | Cephalexin | ≤14 | 14-18 | ≥18 |
|  | Oxacillin | ≤10 | 10-13 | ≥13 |
| Macrolide antibiotics | Mideamycin | ≤13 | 13-18 | ≥18 |
|  | Erythromycin | ≤13 | 13-23 | ≥23 |
| Aminoglycoside antibiotics | Gentamicin | ≤12 | 12-15 | ≥15 |
|  | kanamycin | ≤13 | 13-18 | ≥18 |
|  | Streptomycin | ≤11 | 12-15 | ≥15 |
| Tetracycline antibiotics | minocycline | ≤12 | 12-16 | ≥16 |
|  | Doxycycline | ≤12 | 12-16 | ≥16 |
|  | Tetracycline | ≤14 | 14-19 | ≥19 |
| lincosamide antibiotics | Clindamycin | ≤14 | 14-21 | ≥21 |
| Chloramphenicol antibiotics | Chloramphenicol | ≤12 | 12-18 | ≥18 |
|  | Florfenicol | ≤12 | 12-18 | ≥18 |
| Polypeptide antibiotics | vancomycin | ≤9 | 9-12 | ≥12 |
|  | Bacitracin | ≤8 | 8-13 | ≥13 |
|  | Polymyxin B | ≤8 | 8-12 | ≥12 |
| Quinolone antibiotics | Ciprofloxacin | ≤15 | 15-21 | ≥21 |
|  | Ofloxacin | ≤12 | 12-16 | ≥16 |
| Nitrofuran antibiotics | Furazolidone | ≤14 | 14-17 | ≥17 |
| Sulfonamide antibiotics | Sulfamethoxazole | ≤12 | 12-17 | ≥17 |

**Supplementary Table 2** Strain number, source, toxinotypes, toxin gene, sequence type and clonal complex (n=144).

| Strains ID | Source | Type | Toxin genes | | | | | | | ST | CCs |
| --- | --- | --- | --- | --- | --- | --- | --- | --- | --- | --- | --- |
|  |  |  | *cpa* | *cpb* | *etx* | *iap* | *cpe* | *netB* | *cpb2* |  |  |
| N01 | Qilian County | A | + | - | - | - | - | - | - | 1 |  |
| N02 | Qilian County | A | + | - | - | - | - | - | - | 2 |  |
| N03 | Qilian County | A | + | - | - | - | - | - | - | 3 | CC10 |
| N04 | Qilian County | A | + | - | - | - | - | - | - | 3 | CC10 |
| N05 | Qilian County | A | + | - | - | - | - | - | - | 3 | CC10 |
| N06 | Qilian County | A | + | - | - | - | - | - | - | 3 | CC10 |
| N07 | Qilian County | A | + | - | - | - | - | - | - | 3 | CC10 |
| N08 | Chengduo County | A | + | - | - | - | - | - | - | 4 |  |
| N09 | Chengduo County | A | + | - | - | - | - | - | - | 5 |  |
| N10 | Chengduo County | A | + | - | - | - | - | - | - | 6 |  |
| N11 | Qingshuihe Town | A | + | - | - | - | - | - | - | 7 |  |
| N12 | Qingshuihe Town | F | + | - | - | - | + | - | - | 8 |  |
| N13 | Qingshuihe Town | A | + | - | - | - | - | - | - | 9 |  |
| N14 | Qingshuihe Town | A | + | - | - | - | - | - | - | 10 | CC6 |
| N15 | Qingshuihe Town | A | + | - | - | - | - | - | - | 10 | CC6 |
| N16 | Qingshuihe Town | A | + | - | - | - | - | - | - | 11 |  |
| N17 | Qingshuihe Town | F | + | - | - | - | + | - | - | 12 |  |
| N18 | Qingshuihe Town | A | + | - | - | - | - | - | - | 12 |  |
| N19 | Qingshuihe Town | A | + | - | - | - | - | - | - | 13 | CC1 |
| N20 | Datong County | A | + | - | - | - | - | - | - | 14 |  |
| N21 | Datong County | D | + | - | + | - | - | - | - | 15 | CC7 |
| N22 | Datong County | A | + | - | - | - | - | - | - | 16 |  |
| N23 | Datong County | D | + | - | + | - | - | - | - | 15 | CC7 |
| N24 | Datong County | A | + | - | - | - | - | - | - | 17 |  |
| N25 | Datong County | D | + | - | + | - | - | - | - | 15 | CC7 |
| N26 | Datong County | A | + | - | - | - | - | - | - | 17 |  |
| N27 | Datong County | A | + | - | - | - | - | - | - | 17 |  |
| N28 | Datong County | A | + | - | - | - | - | - | - | 18 |  |
| N29 | Datong County | A | + | - | - | - | - | - | - | 16 |  |
| N30 | Datong County | D | + | - | + | - | - | - | - | 19 | CC7 |
| N31 | Datong County | A | + | - | - | - | - | - | - | 8 |  |
| N32 | Datong County | A | + | - | - | - | - | - | - | 10 | CC6 |
| N33 | Datong County | A | + | - | - | - | - | - | + | 20 |  |
| N34 | Datong County | A | + | - | - | - | - | - | - | 10 | CC6 |
| N35 | Datong County | A | + | - | - | - | - | - | - | 8 |  |
| N36 | Datong County | A | + | - | - | - | - | - | - | 21 |  |
| N37 | Datong County | A | + | - | - | - | - | - | - | 10 | CC6 |
| N38 | Zhiduo County | A | + | - | - | - | - | - | + | 22 |  |
| N39 | Zhiduo County | A | + | - | - | - | - | - | - | 23 |  |
| N40 | Zhiduo County | A | + | - | - | - | - | - | - | 22 |  |
| N41 | Zhiduo County | A | + | - | - | - | - | - | - | 24 | CC11 |
| N42 | Zhiduo County | C | + | + | - | - | - | - | - | 25 |  |
| N43 | Zhiduo County | A | + | - | - | - | - | - | - | 26 | CC11 |
| N44 | Zhiduo County | C | + | + | - | - | - | - | - | 27 |  |
| N45 | Zhiduo County | C | + | + | - | - | - | - | - | 25 |  |
| N46 | Zhiduo County | A | + | - | - | - | - | - | - | 28 |  |
| N47 | Zhiduo County | C | + | + | - | - | - | - | - | 29 |  |
| N48 | Zhiduo County | C | + | + | - | - | - | - | - | 25 |  |
| N49 | Zhiduo County | A | + | - | - | - | - | - | - | 30 | CC3 |
| N50 | Zaduo County | A | + | - | - | - | - | - | - | 31 | CC5 |
| N51 | Zaduo County | A | + | - | - | - | - | - | - | 32 | CC4 |
| N52 | Zaduo County | A | + | - | - | - | - | - | - | 4 |  |
| N53 | Zaduo County | A | + | - | - | - | - | - | - | 33 | CC4 |
| N54 | Zaduo County | A | + | - | - | - | - | - | - | 33 | CC4 |
| N55 | Zaduo County | A | + | - | - | - | - | - | - | 34 | CC10 |
| N56 | Zaduo County | A | + | - | - | - | - | - | - | 33 | CC4 |
| N57 | Zaduo County | A | + | - | - | - | - | - | - | 35 |  |
| N58 | Zaduo County | A | + | - | - | - | - | - | - | 33 | CC4 |
| N59 | Zaduo County | A | + | - | - | - | - | - | - | 4 |  |
| N60 | Zaduo County | A | + | - | - | - | - | - | - | 36 | CC2 |
| N61 | Zaduo County | A | + | - | - | - | - | - | - | 36 | CC2 |
| N62 | Baingoin County | F | + | - | - | - | + | - | - | 37 |  |
| N63 | Baingoin County | A | + | - | - | - | - | - | + | 38 | CC5 |
| N64 | Baingoin County | F | + | - | - | - | + | - | - | 39 |  |
| N65 | Baingoin County | A | + | - | - | - | - | - | - | 40 |  |
| N66 | Baingoin County | A | + | - | - | - | - | - | - | 41 | CC10 |
| N67 | Baingoin County | C | + | + | - | - | - | - | - | 42 |  |
| N68 | Baingoin County | A | + | - | - | - | - | - | - | 43 | CC3 |
| N69 | Baingoin County | C | + | + | - | - | - | - | - | 44 |  |
| N70 | Baingoin County | A | + | - | - | - | - | - | - | 45 |  |
| N71 | Baingoin County | C | + | + | - | - | - | - | - | 41 | CC10 |
| N72 | Baingoin County | A | + | - | - | - | - | - | - | 46 |  |
| N73 | Baingoin County | C | + | + | - | - | - | - | - | 47 | CC8 |
| N74 | Baingoin County | A | + | - | - | - | - | - | + | 48 |  |
| N75 | Baingoin County | A | + | - | - | - | - | - | - | 49 |  |
| N76 | Baingoin County | C | + | + | - | - | - | - | - | 50 |  |
| N77 | Baingoin County | A | + | - | - | - | - | - | - | 51 |  |
| N78 | Baingoin County | A | + | - | - | - | - | - | - | 45 |  |
| N79 | Baingoin County | A | + | - | - | - | - | - | - | 38 | CC5 |
| N80 | Baingoin County | A | + | - | - | - | - | - | - | 52 |  |
| N81 | Baingoin County | A | + | - | - | - | - | - | - | 53 | CC2 |
| N82 | Baingoin County | A | + | - | - | - | - | - | - | 54 |  |
| N83 | Baingoin County | C | + | + | - | - | - | - | - | 55 |  |
| N84 | Lhasa | A | + | - | - | - | - | - | - | 56 | CC9 |
| N85 | Lhasa | A | + | - | - | - | - | - | - | 57 |  |
| N86 | Lhasa | A | + | - | - | - | - | - | - | 58 |  |
| N87 | Lhasa | A | + | - | - | - | - | - | - | 56 | CC9 |
| N88 | Lhasa | A | + | - | - | - | - | - | - | 59 |  |
| N89 | Lhasa | A | + | - | - | - | - | - | - | 60 |  |
| N90 | Lhasa | A | + | - | - | - | - | - | - | 61 | CC1 |
| N91 | Lhasa | A | + | - | - | - | - | - | - | 10 | CC6 |
| N92 | Lhasa | A | + | - | - | - | - | - | - | 56 | CC9 |
| N93 | Lhasa | C | + | + | - | - | - | - | - | 62 | CC9 |
| N94 | Lhasa | C | + | + | - | - | - | - | - | 63 |  |
| N95 | Lhasa | C | + | + | - | - | - | - | - | 63 |  |
| N96 | Shigatse | A | + | - | - | - | - | - | - | 64 |  |
| N97 | Shigatse | A | + | - | - | - | - | - | - | 10 | CC6 |
| N98 | Shigatse | A | + | - | - | - | - | - | - | 10 | CC6 |
| N99 | Shigatse | C | + | + | - | - | - | - | - | 63 |  |
| N100 | Shigatse | A | + | - | - | - | - | - | - | 65 |  |
| N101 | Shigatse | A | + | - | - | - | - | - | - | 66 | CC6 |
| N102 | Shigatse | A | + | - | - | - | - | - | - | 10 | CC6 |
| N103 | Nyingchi | F | + | - | - | - | + | - | - | 67 |  |
| N104 | Nyingchi | F | + | - | - | - | + | - | - | 47 | CC8 |
| N105 | Nyingchi | A | + | - | - | - | - | - | - | 68 |  |
| N106 | Nyingchi | A | + | - | - | - | - | - | - | 69 |  |
| N107 | Nyingchi | A | + | - | - | - | - | - | - | 70 |  |
| N108 | Nyingchi | A | + | - | - | - | - | - | - | 71 |  |
| N109 | Nyingchi | A | + | - | - | - | - | - | - | 72 |  |
| N110 | Nyingchi | A | + | - | - | - | - | - | - | 73 |  |
| N111 | Nyingchi | A | + | - | - | - | - | - | - | 73 |  |
| N112 | Nyingchi | A | + | - | - | - | - | - | - | 74 |  |
| N113 | Nyingchi | A | + | - | - | - | - | - | - | 75 |  |
| N114 | Nyingchi | A | + | - | - | - | - | - | - | 76 |  |
| N115 | Nyingchi | F | + | - | - | - | + | - | - | 77 |  |
| N116 | Qusong County | A | + | - | - | - | - | - | - | 78 |  |
| N117 | Qusong County | C | + | + | - | - | - | - | - | 76 |  |
| N118 | Qusong County | A | + | - | - | - | - | - | - | 79 | CC1 |
| N119 | Qusong County | C | + | + | - | - | - | - | - | 76 |  |
| N120 | Qusong County | C | + | + | - | - | - | - | - | 76 |  |
| N121 | Qusong County | A | + | - | - | - | - | - | - | 80 |  |
| N122 | Qusong County | C | + | + | - | - | - | - | - | 76 |  |
| N123 | Qusong County | A | + | - | - | - | - | - | - | 76 |  |
| N124 | Qusong County | A | + | - | - | - | - | - | - | 76 |  |
| N125 | Qusong County | C | + | + | - | - | - | - | - | 76 |  |
| N126 | Sangri County | A | + | - | - | - | - | - | - | 76 |  |
| N127 | Sangri County | A | + | - | - | - | - | - | - | 76 |  |
| N128 | Sangri County | A | + | - | - | - | - | - | - | 81 | CC6 |
| N129 | Sangri County | C | + | + | - | - | - | - | - | 76 |  |
| N130 | Sangri County | A | + | - | - | - | - | - | - | 76 |  |
| N131 | Sangri County | A | + | - | - | - | - | - | - | 81 |  |
| N132 | Sangri County | A | + | - | - | - | - | - | - | 76 |  |
| N133 | Sangri County | A | + | - | - | - | - | - | - | 81 |  |
| N134 | Sangri County | A | + | - | - | - | - | - | - | 80 |  |
| N135 | Sangri County | C | + | + | - | - | - | - | - | 76 |  |
| N136 | Lhari County | A | + | - | - | - | - | - | - | 82 |  |
| N137 | Lhari County | A | + | - | - | - | - | - | - | 83 |  |
| N138 | Lhari County | A | + | - | - | - | - | - | - | 84 |  |
| N139 | Lhari County | A | + | - | - | - | - | - | - | 85 |  |
| N140 | Lhari County | A | + | - | - | - | - | - | - | 86 | CC8 |
| N141 | Lhari County | C | + | + | - | - | - | - | - | 87 |  |
| N142 | Lhari County | C | + | + | - | - | - | - | - | 88 |  |
| N143 | Lhari County | A | + | - | - | - | - | - | - | 89 |  |
| N144 | Lhari County | C | + | + | - | - | - | - | - | 89 |  |

**Supplementary Table 3** Drug resistance patterns of *Clostridium perfringens* from yaks in Qinghai and Tibet.

| Patterns of drug resistance | No. (%) of drug resistant isolates | | |
| --- | --- | --- | --- |
|  | Qinghai(n=61) | Tibet(n=83) | Total(n=144) |
| **Single** | **0** | **0** | **0** |
| **Double** | **1(1.64)** | **1(1.20)** | **2(1.39)** |
| AGA-SAs | 1(100) | 0 | 1(50) |
| CEP-POL | 0 | 1(100) | 1(50) |
| **Triple** | **10(16.39)** | **15(18.07)** | **25(17.36)** |
| AGA-LIN-SAs | 1(10) | 0 | 1(4) |
| MAC-AGA-SAs | 6(60) | 1(6.67) | 7(28) |
| AGA-POL-SAs | 1(10) | 1(6.67) | 2(8) |
| MAC-POL-SAs | 2(20) | 0 | 2(8) |
| MAC-AGA-POL | 0 | 6(40) | 6(24) |
| CPE-AGA-POL | 0 | 2(13.33) | 2(8) |
| AGA-POL-SAs | 0 | 3(20) | 3(12) |
| MAC-AGA-TET | 0 | 1(6.67) | 1(4) |
| MAC-AGA-FZ | 0 | 1(6.67) | 1(4) |
| **Quadruple** | **16(26.23)** | **26(31.33)** | **42(29.17)** |
| CEP-MAC-AGA-SAs | 2(12.5) | 1(3.85) | 3(7.14) |
| MAC-AGA-POL-SAs | 1(6.25) | 1(3.85) | 2(4.76) |
| MAC-AGA-POL-SAs | 7(43.75) | 17(65.38) | 24(57.14) |
| PEN-AGA-POL-SAs | 3(18.75) | 3(11.54) | 6(14.29) |
| MAC-AGA-LIN-SAs | 1(6.25) | 0 | 1(2.38) |
| CEP-MAC-AGA-POL | 1(6.25) | 4(15.38) | 5(11.90) |
| MAC-AGA-FZ-SAs | 1(6.25) | 0 | 1(2.38) |
| **Quintuple** | **14(22.95)** | **23(27.71)** | **37(25.69)** |
| MAC-AGA-LIN-POL-SAs | 3(21.43) | 1(4.35) | 4(10.81) |
| PEN-MAC-AGA-POL-SAs | 3(21.43) | 6(26.09) | 9(24.32) |
| PEN-MAC-AGA-CAP-SAs | 1(7.14) | 2(8.70) | 3(8.11) |
| MAC-AGA-TET-POL-SAs | 3(21.43) | 0 | 3(8.11) |
| MAC-AGA-POL-QUIN-SAs | 1(7.14) | 3(13.04) | 4(10.81) |
| CEP-AGA-CAP-POL-SAs | 2(14.29) | 1(4.35) | 3(8.11) |
| MAC-TET-POL-FZ-SAs | 1(7.14) | 6(7.23) | 7(18.92) |
| CEP-AGA-TET-POL-ASs | 0 | 1(4.35) | 1(2.70) |
| PEN-MAC-AGA-TET-SAs | 0 | 1(4.35) | 1(2.70) |
| MAC-AGA-CAP-POL-SAs | 0 | 2(8.70) | 2(5.41) |
| **Sextuple** | **12(19.67)** | **9(10.84)** | **21(14.58)** |
| PEN-MAC-AGA-POL-FZ-SAs | 3(25) | 1(11.11) | 4(19.05) |
| CPE-MAC-AGA-CAP-POL-SAs | 1(8.33) | 0 | 1(4.76) |
| MAC-AGA-LIN-CAP-POL-SAs | 1(8.33) | 0 | 1(4.76) |
| MAC-AGA-TET-POL-QUIN-SAs | 1(8.33) | 2(22.22) | 3(14.29) |
| PEN-MAC-POL-QUIN-FZ-SAs | 1(8.33) | 0 | 1(4.76) |
| CEP-MAC-AGA-TET-FZ-SAs | 2(16.67) | 0 | 2(9.52) |
| MAC-AGA-POL-QUIN-FZ-SAs | 1(8.33) | 0 | 1(4.76) |
| MAC-AGA-LIN-POL-FZ-SAs | 1(8.33) | 3(33.33) | 4(19.05) |
| PEN-MAC-AGA-LIN-POL-SAs | 1(8.33) | 2(22.22) | 3(14.29) |
| MAC-AGA-TET-LIN-QUIN-SAs | 0 | 1(22.22) | 1(4.76) |
| **Septuple** | **6(9.84)** | **4(4.82)** | **10(6.94)** |
| MAC-AGA-TET-LIN-CAP-POL-SAs | 2(33.33) | 0 | 2(20) |
| CEP-MAC-AGA-LIN-POL-QUIN-SAs | 2(33.33) | 1(25) | 3(30) |
| PEN-AGA-LIN-CAP-POL-FZ-SAs | 1(16.67) | 0 | 1(10) |
| PEN-MAC-AGA-TET-LIN-POL-SAs | 1(16.67) | 0 | 1(10) |
| CEP-MAC-AGA-CAP-POL-FZ-SAs | 0 | 2(50) | 2(20) |
| CEP-MAC-AGA-CAP-POL-QUIN-SAs | 0 | 1(25) | 1(10) |
| **Octuple** | **2(3.28)** | **2(2.41)** | **4(2.78)** |
| PEN-MAC-AGA-TET-POL-QUIN-FZ-SAs | 1(50) | 0 | 1(25) |
| CEP-MAC-AGA-TET-CAP-POL-QUIN-SAs | 1(50) | 2(100) | 3(75) |
| **Nonuple** | **0** | **2(2.41)** | **2(1.39)** |
| PEN-MAC-AGA-TET-LIN-CAP-POL-FZ-SAs | 0 | 1(50) | 1(50) |
| PEN-MAC-AGA-TET-LIN-POL-QUIN-FZ-SAs | 0 | 1(50) | 1(50) |

**Notes:** PEN, Penicillin antibiotics; CEP, Cephalosporin antibiotics; MAC, Macrolide antibiotics; AGA, Aminoglycoside antibiotics; TET, Tetracycline antibiotics; LIN, lincosamide antibiotics; CAP, Chloramphenicol antibiotics; POL, Polypeptide antibiotics; QUIN, Quinolone antibiotics; FZ, Furazolidone antibiotics; SAs, Sulfonamide antibiotics; PEN and CEP belong to β-Lactam antibiotics.
